# Clinical features and diagnostic pathways of trigeminal neuralgia: a retrospective study in a tertiary orofacial pain clinic

**DOI:** 10.22514/jofph.2026.043

**Published:** 2026-05-12

**Authors:** Thaviporn Limrachtamorn

**Affiliations:** ^1^Faculty of Dentistry, Thammasat University, 12120 Pathum Thani, Thailand

**Keywords:** Trigeminal neuralgia, Orofacial pain, Neuropathic pain, Clinical features, Diagnostic pathways, Retrospective study

## Abstract

**Background**: Trigeminal neuralgia (TN) is a neuropathic facial pain disorder that frequently mimics odontogenic and other orofacial pain conditions, leading many patients to initially seek dental care. This study aimed to characterize the clinical features and diagnostic pathways of patients with TN presenting to a tertiary orofacial pain clinic. **Methods**: This single-center retrospective study included newly diagnosed patients with TN who attended a tertiary orofacial pain clinic between 2024 and 2025. Data regarding their demographic characteristics, pain features, pain intensity, trigger factors, and trigeminal nerve distribution were collected, and their psychological characteristics were assessed using the Patient Health Questionnaire-9 (PHQ-9), the Generalized Anxiety Disorder-7 (GAD-7), and the 10-item Perceived Stress Scale (PSS-10). Diagnostic pathways before referral, including dental procedures performed before diagnosis, were also evaluated. **Results**: A total of 36 patients were assessed (mean age 66.8 ± 10.7 years; 66.7% female), and most presented with severe paroxysmal electric shock-like pain, predominantly involving the maxillary and mandibular divisions of the trigeminal nerve. Pain episodes were found to be triggered by light facial touch, with additional common triggers including washing the face, tooth brushing, speaking, and chewing. Pain intensity at presentation was graded as severe, with a mean Numerical Rating Scale (NRS) score of 8.17 ± 1.08. Moderate to moderately severe depressive symptoms, moderate to severe anxiety, and moderate perceived stress were frequently observed. Notably, before referral, 22.2% of patients had undergone tooth extraction, and 2.8% had received root canal treatment without achieving pain relief. **Conclusions**: Patients with TN presenting to a tertiary orofacial pain clinic exhibit characteristic neuropathic pain features, although their diagnostic pathways are variable, and a notable proportion undergo dental procedures before a confirmed diagnosis. Better recognition of these characteristic neuropathic pain features may facilitate earlier suspicion of TN, promote timely referral, and reduce unnecessary dental procedures.

## 1. Introduction

Trigeminal neuralgia (TN) is classified in the International Classification of Headache Disorders, 3rd edition (ICHD-3), as a neuropathic facial pain disorder characterized by recurrent, unilateral, brief episodes of facial pain occurring within the distribution of one or more divisions of the trigeminal nerve [1, 2]. These attacks typically present as brief electric shock-like, stabbing, shooting, or sharp pain, with a sudden onset and abrupt cessation, and are frequently triggered by ordinarily non-painful stimuli. In terms of anatomical distribution, TN most commonly involves the maxillary (V2) and mandibular (V3) divisions of the trigeminal nerve, with right-sided involvement reported more frequently than left-sided involvement. In contrast, bilateral presentation remains uncommon and should prompt consideration of a possible secondary cause. From an etiological perspective, TN is classified into classical, secondary, and idiopathic forms. Among these, classical TN is generally associated with neurovascular compression of the trigeminal nerve root, whereas secondary TN arises from identifiable underlying conditions such as multiple sclerosis or space-occupying lesions, and idiopathic TN is diagnosed when no structural cause can be identified despite appropriate evaluation [1, 2]. Epidemiological evidence further indicates that TN occurs more frequently in women than in men and demonstrates an increasing incidence with advancing age. The condition remains rare in children, and onset before the age of 40 years should raise suspicion of a secondary etiology [3, 4]. The reported incidence rates range from approximately 4 to 27 new cases per 100,000 individuals annually, with population-based studies estimating a lifetime prevalence of approximately 0.16–0.3% [5]. Clinically, TN is associated with substantial impairment in quality of life, as it disrupts routine activities such as facial contact, speaking, eating, and drinking, and is further accompanied by a considerable psychological and social burden, reflected by increased levels of anxiety, depressive symptoms, and sleep disturbance among affected individuals [6, 7, 8, 9].

Although TN is traditionally managed within neurology and neurosurgical disciplines, patients frequently first present to dental services because the pain often mimics odontogenic or other orofacial pain conditions [3, 4]. From an orofacial pain perspective, TN represents a prototypical neuropathic facial pain disorder that poses a challenge to conventional dental diagnostic frameworks. Pain localized to the maxillary or mandibular regions, particularly when triggered by chewing, speaking, or light facial contact, may closely resemble dental pathology, atypical odontalgia, or temporomandibular disorders [5, 6]. Despite the availability of established diagnostic criteria, heterogeneity in clinical presentation and overlap with other orofacial pain conditions may lead to diagnostic uncertainty [10]. As a result, patients with TN often undergo extensive dental evaluations or interventions before an accurate diagnosis is established, thereby increasing diagnostic complexity and delaying appropriate management [6, 11]. This diagnostic overlap highlights the important role of dentists in the early recognition of TN. In the field of orofacial pain, the International Classification of Orofacial Pain (ICOP) provides a standardized diagnostic framework for pain conditions affecting the orofacial region, in which TN is categorized as pain attributed to a lesion or disease of the trigeminal nerve [12].

Most existing studies on TN have been derived from neurology-based populations and have primarily focused on treatment outcomes, pharmacological management, or neuroimaging findings [7]. Although these investigations have substantially advanced current understanding of the pathophysiology and management of TN, they provide only limited insight into how patients with TN initially present and are evaluated within dental-based orofacial pain services. In tertiary orofacial pain clinics, patients with TN often present after prolonged and heterogeneous clinical trajectories, during which they may have consulted multiple healthcare providers or undergone various dental procedures before a definitive diagnosis is established [3, 4, 5, 6]. However, systematic data describing the clinical presentation and diagnostic pathways of TN from a dental-based orofacial pain perspective remain limited.

A clearer understanding of the clinical features and diagnostic pathways of TN in tertiary orofacial pain clinic settings is therefore of particular relevance to dental practitioners and orofacial pain specialists. Specifically, characterization of pain features, trigger factors, and referral patterns may facilitate earlier recognition of TN and improve its differentiation from odontogenic and other non-neuropathic orofacial pain conditions. Accordingly, the present study aimed to describe the clinical features and diagnostic pathways of patients diagnosed with TN who presented to a tertiary orofacial pain clinic.

## 2. Materials and methods

### 2.1 Study design and participants

This retrospective review of clinical records was conducted at the Orofacial Pain Clinic, a tertiary referral service of the Faculty of Dentistry, Thammasat University, from 01 January 2024, to 31 December 2025. Ethical approval was granted by the Human Research Ethics Committee of Thammasat University (Science), Thailand (Project No. 68DE167; Certificate of Exemption No. 002/2569). The study was conducted in accordance with internationally recognized ethical principles, including the Declaration of Helsinki, the Belmont Report, and the International Conference on Harmonisation-Good Clinical Practice (ICH-GCP) guidelines. As the analysis relied exclusively on retrospectively collected clinical records that had been fully anonymized before analysis, the requirement for informed consent was waived.

The inclusion criteria were adult patients (≥18 years) who presented to the clinic for the first time and received a confirmed diagnosis of TN at the clinic according to the ICHD-3 criteria. The exclusion criteria were incomplete medical records and a history of head and neck cancer. The ICHD-3 diagnostic criteria applied in this study are summarized in Table 1. Medical records that fulfilled all inclusion criteria and none of the exclusion criteria were retrieved from the clinic database. The extracted variables included demographic characteristics (age and sex); pain characteristics (electric shock-like, shooting, stabbing, or sharp pain); pain intensity assessed using the Numerical Rating Scale (NRS); onset duration (in months) and onset category (≤3 months or >3 months); duration of pain per attack (in seconds); pain location; pain laterality; and trigeminal nerve division involvement recorded separately for the right and left sides. Trigger factors, including mechanical and functional stimuli, were also recorded. Psychological characteristics were evaluated using validated screening instruments, namely the Patient Health Questionnaire-9 (PHQ-9), the Generalized Anxiety Disorder-7 (GAD-7), and the 10-item Perceived Stress Scale (PSS-10), and were recorded as both raw scores and categorized severity levels. Variables related to the diagnostic pathway included the type of clinician consulted before presentation, previous diagnostic impressions documented in the medical history, and a history of dental procedures performed before referral and prior to a confirmed diagnosis of TN.

**Table 1.** Diagnostic criteria for TN according to the ICHD-3.

Criterion	Description
A	Recurrent paroxysms of unilateral facial pain in the distribution(s) of one or more divisions of the trigeminal nerve, with no radiation beyond, and fulfilling criteria B and C
B	Pain with all the following characteristics: 1. Lasting from a fraction of a second to two minutes 2. Severe intensity, and 3. Electric shock-like, shooting, stabbing, or sharp in quality
C	Precipitated by innocuous stimuli within the affected trigeminal distribution
D	Not better accounted for by another ICHD-3 diagnosis

*ICHD-3: International Classification of Headache Disorders, 3rd edition.*

The study was conducted and reported in accordance with the Strengthening the Reporting of Observational Studies in Epidemiology (STROBE) guidelines. A flow diagram summarizing the numbers of patients screened, included, and excluded, together with the reasons for exclusion, is provided in Fig. 1. All study variables were defined before data extraction, and the analyses were performed based on the predefined study objectives and a descriptive analytic approach. Given the retrospective design of the study, no a priori sample size determination or formal power calculation was performed.

**Fig. 1.** STROBE-compliant participant flow diagram showing the number of patients screened, excluded (with reasons), and included in the final analysis.

### 2.2 Data collection and examiner

All clinical assessments and diagnostic determinations during routine care, as well as the retrospective extraction of data and verification against the original charts, were performed by the same staff specialist in orofacial pain. The diagnosis of TN was established during routine clinical evaluation according to the ICHD-3 diagnostic criteria (Table 1). In all included cases, this diagnosis represented the patient’s first confirmed diagnosis of TN. To ensure diagnostic consistency, the examiner followed a standardized assessment approach that is routinely applied in the clinic.

In this retrospective study, patients were not systematically categorized into etiological subtypes of TN, such as classical, secondary, or idiopathic. In addition, neuroimaging examinations, such as magnetic resonance imaging (MRI), were not routinely performed within the dental clinic. When clinically indicated for the exclusion of potential secondary causes of TN, patients were referred to medical services, primarily neurology, for further evaluation and imaging. Clinical information documented in the narrative clinical notes was reviewed and categorized into predefined study variables, including pain descriptors, trigger factors, anatomical pain locations, and prior diagnostic impressions. All study variables were defined before data extraction. When multiple descriptions were recorded in the clinical notes, the predominant description documented during the initial consultation was used. Although no formal standardized data extraction form was used, data were extracted using an informal structured template used within the clinic, along with a predefined set of variables based on the study protocol to ensure consistent and systematic data abstraction.

### 2.3 Measures

#### 2.3.1 Pain characteristics

Pain characteristics were extracted from the medical records based on patient-reported descriptors documented during the initial clinical evaluation. Pain quality was categorized as electric shock-like, shooting, stabbing, or sharp pain, in accordance with descriptors commonly reported in TN. These variables were used to characterize the predominant clinical presentation of pain.

#### 2.3.2 Pain intensity

Pain intensity was assessed using the Numerical Rating Scale (NRS), on which patients rated their current pain on a scale from 0 (no pain) to 10 (worst imaginable pain). NRS scores were analyzed as continuous variables and, for descriptive purposes, were additionally grouped into mild (1–3), moderate (4–6), and severe (7–10) categories according to established pain severity classifications.

#### 2.3.3 Onset duration

Symptom duration before presentation was calculated from the patient-reported time of symptom onset to the first clinic visit and was recorded in months. For descriptive purposes, symptom duration was additionally categorized as ≤3 months or >3 months.

#### 2.3.4 Duration of pain in each attack

The duration of pain in each attack was recorded in seconds based on patient-reported estimates documented in the medical records. This variable was used to characterize the paroxysmal nature of pain episodes typical of TN.

#### 2.3.5 Location of pain

The anatomical location of pain was documented according to the patient-reported pain distribution, including the teeth, gingiva, temporal area, chin, lip, nose, eye, forehead, cheek, or ear. These locations were recorded descriptively to reflect the variability in pain presentation.

#### 2.3.6 Pain laterality and trigeminal nerve distribution

Involvement of the trigeminal nerve divisions was recorded separately for the right and left sides and was categorized as the ophthalmic (V1), maxillary (V2), and mandibular (V3) divisions, or combinations thereof, based on the anatomical distribution of pain reported by the patient and documented during clinical assessment.

#### 2.3.7 Trigger factors

Trigger factors were recorded based on patient-reported stimuli that precipitated pain attacks, including mechanical or functional triggers, such as facial touch, chewing, brushing teeth, washing the face, speaking, and other activities documented in the medical records.

#### 2.3.8 Psychological measures

Psychological characteristics were evaluated using validated Thai-language versions of standardized self-report questionnaires. In the primary analyses, questionnaire scores were analyzed as continuous variables to preserve statistical sensitivity. For descriptive analyses, the scores were further classified into severity categories according to established threshold values.

##### 2.3.8.1 Depression

Depressive symptoms were assessed using the PHQ-9 [13]. Scores were classified as minimal (0–4), mild (5–9), moderate (10–14), moderately severe (15–19), or severe (20–27). The Thai version of the PHQ-9 has demonstrated acceptable reliability, with a reported Cronbach’s alpha of 0.79 [14].

##### 2.3.8.2 Anxiety

Anxiety symptoms were assessed using the GAD-7 [15] and classified as minimal (0–4), mild (5–9), moderate (10–14), or severe (15–21). The Thai version of the GAD-7 has demonstrated high reliability, with a reported Cronbach’s alpha of 0.89 [16].

##### 2.3.8.3 Perceived stress

Perceived stress was assessed using the 10-item PSS-10 [17]. Scores were classified as low (0–13), moderate (14–26), or high (27–40). The Thai version of the PSS-10 has demonstrated good reliability, with a reported Cronbach’s alpha of 0.85 [18].

#### 2.3.9 Diagnostic pathway variables

Diagnostic pathway variables included the type of clinician consulted prior to presentation at the orofacial pain clinic, previous diagnostic impressions documented in the medical history, and a history of dental procedures performed prior to referral and before a confirmed diagnosis of TN.

### 2.4 Statistical analysis

All statistical analyses were performed using IBM SPSS Statistics, version 26.0 (IBM Corp., Armonk, NY, USA). Given the exploratory and descriptive nature of this retrospective study, the analyses were primarily descriptive. Continuous variables are summarized as mean ± standard deviation (SD) when approximately normally distributed and as median with interquartile range (IQR) when non-normally distributed; minimum and maximum values are also reported where relevant. Categorical data are presented as frequencies and percentages.

Variables that allowed multiple responses per patient (*e.g.*, pain locations and trigger factors) were coded as binary indicators (present/absent) and summarized as counts and percentages; therefore, totals may exceed 100%. Trigeminal nerve division patterns were derived from the recorded involvement of V1, V2, and V3 on each side and were categorized as V2 only, V3 only, or combined V2 and V3 involvement. Pain intensity, assessed using the NRS (0–10), was analyzed as a continuous variable and additionally categorized as mild (1–3), moderate (4–6), or severe (7–10) for descriptive reporting. Psychological measures, including the PHQ-9, GAD-7, and PSS-10, were reported using both raw scores and established severity categories.

Complete data were available for all variables included in the analyses; therefore, no data imputation was required. No a priori sample size calculation was undertaken, and no inferential hypothesis testing or multivariable modeling was performed.

## 3. Results

### 3.1 Demographic characteristics

A total of 36 patients with TN were included in the study. The mean age was 66.8 ± 10.7 years, with a range of 51–91 years. Female patients accounted for 66.7% (n = 24) of the study population, and 12 patients (33.3%) were male. The demographic characteristics of the study population are summarized in Table 2.

**Table 2.** Demographic and clinical characteristics (N = 36).

Variables		Value
Age (yr), mean ± SD (range)		66.8 ± 10.7 (51–91)
Sex		
	Female, n (%)	24 (66.7)
	Male, n (%)	12 (33.3)
Pain intensity (NRS)		
	Mean ± SD	8.17 ± 1.08
	Median (IQR), range	8 (7–9), 7–10
Symptom duration prior to presentation (mon)		
	Median (IQR), range	7.5 (5–36), 4–120
	Onset >3 mon, n (%)	36 (100.0)
Duration of pain in each attack (seconds)		
	Median (IQR), range	60 (30–60), 30–120

*SD: standard deviation; IQR: interquartile range; NRS: Numerical Rating Scale.*

### 3.2 Pain characteristics

Electric shock-like pain was the most frequently reported pain quality and was documented in 25 patients (69.4%). Other reported pain qualities included stabbing pain in 5 patients (13.9%), and shooting pain and sharp pain in 3 patients each (8.3%). The pain characteristics are summarized in Table 3.

**Table 3.** Pain characteristics (N = 36).

Pain characteristics	n (%)
Electric shock-like	25 (69.4)
Stabbing	5 (13.9)
Shooting	3 (8.3)
Sharp	3 (8.3)

*Percentages may not total 100% due to rounding.*

### 3.3 Pain intensity

Pain intensity at the time of presentation was within the severe range. The mean pain intensity score was 8.17 ± 1.08, and the median score was 8 (IQR, 7–9; range, 7–10). Pain intensity data are summarized in Table 2.

### 3.4 Onset duration

The median duration from symptom onset to referral was 7.5 months (interquartile range (IQR), 5–36 months), with a wide range of 4–120 months. All patients had experienced pain for longer than 3 months at the time of referral. Onset duration data are summarized in Table 2.

### 3.5 Duration of pain in each attack

The median duration of pain in each attack was 60 seconds (IQR, 30–60 seconds), with reported durations ranging from 30 to 120 seconds. The duration of pain in each attack is summarized in Table 2.

### 3.6 Location of pain

Pain was most commonly reported in the cheek (61.1%), gingiva (58.3%), and teeth (55.6%). Other frequently involved sites included the temporal region and nasal ala, each of which was reported in 41.7% of patients. Less common pain locations included the lateral canthus (25.0%), chin (19.4%), and lip (19.4%). Individual patients often reported multiple pain locations and are summarized in Table 4.

**Table 4.** Pain location (multiple responses were allowed) (N = 36).

Location	n (%)
Cheek	22 (61.1)
Gingiva	21 (58.3)
Teeth	20 (55.6)
Temporal region	15 (41.7)
Nose (ala)	15 (41.7)
Lateral canthus	9 (25.0)
Chin	7 (19.4)
Lip	7 (19.4)

*Multiple pain locations could be reported by individual patients.*

### 3.7 Pain laterality and trigeminal nerve distribution

Pain was predominantly right-sided and occurred in 23 patients (63.9%), followed by left-sided involvement in 12 patients (33.3%). Bilateral symptoms were observed in 1 patient (2.8%). Combined involvement of the V2 and V3 divisions was the most common pattern of trigeminal nerve distribution. In contrast, isolated involvement of V2 or V3 occurred less frequently, and no involvement of V1 was identified. The distribution of trigeminal nerve involvement by side is summarized in Table 5.

**Table 5.** Trigeminal nerve division pattern by side (N = 36).

Trigeminal division pattern	Right n (%)	Left n (%)	Bilateral n (%)	Total n (%)
V2 only	4 (11.1)	4 (11.1)	0 (0.0)	8 (22.2)
V3 only	3 (8.3)	3 (8.3)	0 (0.0)	6 (16.7)
V2 and V3	16 (44.4)	5 (13.9)	1 (2.8)	22 (61.1)
V1 involvement (any pattern)	0 (0.0)	0 (0.0)	0 (0.0)	0 (0.0)
Total	23 (63.9)	12 (33.3)	1 (2.8)	36 (100.0)

*V1: ophthalmic division; V2: maxillary division; V3: mandibular division of the trigeminal nerve. Trigeminal nerve involvement was recorded separately for the right and left sides. Patients could present with involvement of more than one trigeminal nerve division.*

### 3.8 Trigger factors

All patients reported pain triggered by light facial touch. Other commonly reported triggers included washing the face (66.7%), brushing the teeth (44.4%), speaking (38.9%), and chewing (36.1%). Less frequently reported triggers included shaving (22.2%) and exposure to air drafts (16.7%). The trigger factors are summarized in Table 6.

**Table 6.** Trigger factors (multiple responses were allowed) (N = 36).

Trigger	n (%)
Touching the face	36 (100.0)
Washing the face	24 (66.7)
Tooth brushing	16 (44.4)
Speaking activities	14 (38.9)
Chewing	13 (36.1)
Shaving	8 (22.2)
Exposure to air drafts	6 (16.7)

*Multiple trigger factors could be reported by individual patients.*

### 3.9 Psychological characteristics

Complete data were available for all 36 patients for the PHQ-9, GAD-7, and PSS-10 (n = 36 for each instrument). The mean PHQ-9 score was 13.7 ± 4.2, indicating that depressive symptoms were predominantly within the moderate to moderately severe range. The mean GAD-7 score was 13.5 ± 3.6, reflecting that most patients experienced moderate to severe anxiety. The mean PSS-10 score was 21.1 ± 6.0, corresponding primarily to moderate levels of perceived stress. The detailed psychological characteristics are presented in Table 7.

**Table 7.** Psychological characteristics (N = 36).

Measure	Summary score	Severity category	n (%)
PHQ-9			
	Mean ± SD: 13.7 ± 4.2	Minimal (0–4)	1 (2.8)
	Median (IQR): 14 (10–18)	Mild (5–9)	3 (8.3)
	Range: 4–20	Moderate (10–14)	17 (47.2)
		Moderately severe (15–19)	14 (38.9)
		Severe (20–27)	1 (2.8)
GAD-7			
	Mean ± SD: 13.5 ± 3.6	Minimal (0–4)	1 (2.8)
	Median (IQR): 14 (11–16)	Mild (5–9)	4 (11.1)
	Range: 4–20	Moderate (10–14)	18 (50.0)
		Severe (15–21)	13 (36.1)
PSS-10			
	Mean ± SD: 21.1 ± 6.0	Low (0–13)	5 (13.9)
	Median (IQR): 22 (14–27)	Moderate (14–26)	21 (58.3)
	Range: 12–30	High (27–40)	10 (27.8)

*SD: standard deviation; IQR: interquartile range; PHQ-9: Patient Health Questionnaire-9; GAD-7: Generalized Anxiety Disorder-7; PSS-10: 10-item Perceived Stress Scale. Severity categories are based on established cut-off scores for each instrument. Complete data were available for all 36 patients for the PHQ-9, GAD-7, and PSS-10 (n: 36 for each instrument).*

### 3.10 Diagnostic pathway prior to referral

Regarding the diagnostic pathway, all patients initially sought care from general dentists in private dental practices before referral to the orofacial pain clinic. Eight patients (22.2%) had undergone tooth extraction, and one patient (2.8%) had received root canal treatment before referral. These dental procedures were performed before a confirmed diagnosis of TN had been established and were based on the clinical presentation at that time.

Of the nine patients who underwent dental procedures, seven had diagnostic impressions related to dental caries, and two had diagnostic impressions related to periodontal conditions, as documented in the available medical records. In all cases, these dental interventions did not result in pain relief, and patients continued to experience persistent symptoms following treatment.

## 4. Discussion

This retrospective study describes the clinical characteristics and diagnostic pathways of patients with TN presenting to a tertiary orofacial pain clinic. The demographic profile of the study population, characterized by a mean age in the late sixth to seventh decade and a female predominance of approximately two-thirds, is consistent with previous epidemiological evidence indicating that TN occurs more frequently in older individuals and affects women more often than men [7, 19, 20]. Among the reported pain characteristics, electric shock-like pain was the most common feature, followed by stabbing, shooting, and sharp pain, which is consistent with the neuropathic pain phenotype defined by the ICHD-3 [1]. Pain intensity at presentation was observed to be within the severe range, with mean NRS scores exceeding 8, underscoring the substantial pain burden at the time of referral. Consistent with previous reports, TN is recognized as one of the most severe pain conditions in clinical practice and may lead to repeated healthcare consultations before a diagnosis is established [21]. All patients reported symptom duration of more than three months, and in many cases the duration extended over several years, highlighting the frequently prolonged diagnostic trajectory associated with TN [21]. Comparatively, the duration of individual pain attacks remained brief, typically lasting from seconds to minutes, reinforcing the paroxysmal profile characteristic of TN. This dissociation between prolonged disease duration and brief pain attacks may contribute to diagnostic uncertainty, particularly in dental settings, where episodic pain may be more often attributed to localized odontogenic pathology rather than an underlying neuropathic mechanism.

Pain was most commonly reported in the cheek, gingiva, and teeth, with predominant involvement of the V2 and V3 divisions and a marked right-sided predominance. These findings are consistent with established clinical evidence showing that TN most commonly involves the V2 and V3 divisions and occurs more frequently on the right side [7]. The frequent involvement of intraoral and dentoalveolar regions provides a plausible explanation for why TN may initially be misinterpreted as odontogenic pain. In addition, triggerability was a universal feature, as all patients reported pain precipitated by light facial touch, and many also described additional triggers such as washing the face, tooth brushing, speaking, and chewing. The predominance of paroxysmal electric shock-like pain, together with stimulus-triggered attacks, aligns with with previously reported clinical phenotypes of TN, including the study by Haviv *et al*. [22]. Taken together, these findings underscore the importance of systematically assessing pain timing, anatomical distribution, pain quality, pain severity, and triggers when differentiating odontogenic from non-odontogenic orofacial pain in dental practice [23].

Beyond the physical characteristics of pain, patients in this study also demonstrated a substantial psychological burden, with predominantly moderate to moderately severe depressive symptoms, moderate to severe anxiety, and moderate levels of perceived stress. These observations are broadly consistent with previous literature describing the psychological burden associated with TN [24, 25]. In the present study, psychological measures were included to characterize the psychological burden of the study population rather than to examine causal relationships with specific pain characteristics. Accordingly, these findings should be interpreted descriptively and with caution, particularly in the absence of a control group or longitudinal follow-up.

A key finding of this study was the heterogeneity of diagnostic pathways before presentation at the tertiary orofacial pain clinic. A notable proportion of patients had undergone dental procedures, including tooth extraction and endodontic treatment, before a definitive diagnosis of TN was established. These interventions likely reflect symptomatic overlap between TN and odontogenic pain, as well as the diagnostic complexity of neuropathic facial pain in dental practice [3, 7]. Similar patterns have been previously reported, with many patients undergoing irreversible dental procedures without pain relief before the correct diagnosis [26, 27]. Such heterogeneous and often prolonged diagnostic pathways may contribute to patient distress and uncertainty before an accurate diagnosis is made [28]. Within the present dataset, findings directly support that some patients underwent dental procedures before referral and before a confirmed diagnosis of TN. Therefore, broader interpretations regarding diagnostic delay should be regarded as contextual observations rather than direct outcomes of this study. Survey-based studies have also reported confusion between TN and odontogenic pain among dentists and have emphasized the importance of familiarity with diagnostic criteria to reduce unnecessary dental interventions [29]. Collectively, these findings highlight the importance of recognizing characteristic neuropathic pain features in dental practice to facilitate earlier suspicion of TN and appropriate referral.

Although treatment outcomes were beyond the scope of the present retrospective analysis, routine management of TN at the tertiary orofacial pain clinic typically begins after diagnosis is established. Pharmacological therapy, most commonly carbamazepine, may be prescribed as first-line treatment in accordance with established clinical recommendations. In our clinical setting, carbamazepine is not used as a diagnostic test. Given the well-recognized risk of severe cutaneous adverse reactions associated with carbamazepine in Asian populations, HLA-B*15:02 screening is routinely performed before treatment initiation. Patients may subsequently be referred to neurology services for further neurological evaluation, neuroimaging, or more advanced management when clinically indicated. Medication response and long-term outcomes were not systematically collected and were not analyzed in this study.

Limitations of this study include its retrospective design, modest sample size, and reliance on routine clinical records. Patients were not systematically categorized into etiological subtypes of TN, namely classical, secondary, or idiopathic, and neuroimaging examinations such as MRI were not uniformly available, as imaging was obtained primarily through referral to neurology when clinically indicated to secondary causes. Standardized neuropathic pain questionnaires, such as the Douleur Neuropathique en 4 Questions (DN4) or Leeds Assessment of Neuropathic Symptoms and Signs (LANSS), were not systematically used, and autonomic symptoms were not consistently documented, limiting detailed phenotypic characterization. Additionally, detailed information regarding earlier stages of the diagnostic pathway before referral to the tertiary clinic, including the number of clinicians consulted before diagnosis and prior pharmacological treatments, was not consistently documented. All patients presented with symptom duration exceeding three months, reflecting the tertiary referral nature of the clinic, which may limit the generalizability to earlier-stage TN presentations. As a single-center study conducted in a tertiary referral setting, the study population may represent patients with more advanced or persistent symptoms. Moreover, because this study is descriptive any interpretation of associations between psychological findings and pain characteristics should be made with caution. Nevertheless, the present findings provide clinically relevant insights into the clinical features and diagnostic pathways of TN in a tertiary dental-based orofacial pain service.

## 5. Conclusions

This retrospective study conducted in a Thai tertiary orofacial pain clinic demonstrated that patients with TN commonly presented with severe paroxysmal electric shock-like pain, predominantly involving the maxillary and mandibular divisions and triggered by innocuous stimuli. The diagnostic pathways were heterogeneous, and a notable proportion of patients had undergone dental procedures before a confirmed diagnosis. These findings underscore the importance of recognizing the characteristic neuropathic pain features of TN in dental practice, to support earlier clinical suspicion, facilitate timely referral, and reduce potentially unnecessary dental interventions.
